# Granulocyte colony-stimulating factor blockade enables dexamethasone to inhibit lipopolysaccharide-induced murine lung neutrophils

**DOI:** 10.1371/journal.pone.0177884

**Published:** 2017-05-19

**Authors:** Jesus Banuelos, Yun Cao, Soon Cheon Shin, Bruce S. Bochner, Pedro Avila, Shihong Li, Xin Jiang, Mark W. Lingen, Robert P. Schleimer, Nick Z. Lu

**Affiliations:** 1 Division of Allergy-Immunology, Feinberg School of Medicine, Northwestern University, Chicago, Illinois, United States of America; 2 Department of Pharmacology and Human Tissue Resource Center, The University of Chicago, Chicago, Illinois, United States of America; Cedars-Sinai Medical Center, UNITED STATES

## Abstract

Glucocorticoids promote neutrophilic inflammation, the mechanisms of which are poorly characterized. Using a lipopolysaccharide (LPS)-induced acute murine lung injury model, we determined the role of granulocyte colony-stimulating factor (G-CSF) in mouse lung neutrophil numbers in the absence and presence of dexamethasone, a potent glucocorticoid. G-CSF was blocked using a neutralizing antibody. Airway neutrophil numbers, cytokine levels, and lung injury parameters were measured. Glucocorticoid treatment maintained LPS-induced airway G-CSF while suppressing TNF and IL-6. The addition of anti-G-CSF antibodies enabled dexamethasone to decrease airway G-CSF, neutrophils, and lung injury scores. In LPS-challenged murine lungs, structural cells and infiltrating leukocytes produced G-CSF. *In vitro* using BEAS 2B bronchial epithelial cells, A549 lung epithelial cells, human monocyte-derived macrophages, and human neutrophils, we found that dexamethasone and proinflammatory cytokines synergistically induced G-CSF. Blocking G-CSF production in BEAS 2B cells using shRNAs diminished the ability of BEAS 2B cells to protect neutrophils from undergoing spontaneous apoptosis. These data support that G-CSF plays a role in upregulation of airway neutrophil numbers by dexamethasone in the LPS-induced acute lung injury model.

## Introduction

Neutrophils are the most abundant leukocytes in the circulation and they play a pathogenic role in various inflammatory conditions [1]. Signals that trigger proinflammatory responses of neutrophils include damage-associated molecular patterns, pathogen-associated molecular patterns, and proinflammatory stimuli generated by adaptive immunity (e.g., IFNγ and IL-17) [1]. Although it is well-documented that the proinflammatory processes mediated by neutrophils are necessary for host defense, activated neutrophils produce reactive oxygen species (ROS) and proteases that can damage host tissues [1]. Therefore, it is necessary to limit inflammation overshoot in diseases where neutrophilic responses are exaggerated.

Glucocorticoids are widely used antiinflammatory agents. Repressing proinflammatory cytokines, stimulating anti-inflammatory genes, and promoting apoptosis of selective groups of leukocytes (eosinophils, T cells, and mature dendritic cells) are some of the major pathways by which glucocorticoids block excessive inflammation [2–6]. Surprisingly, glucocorticoids also enhance innate immunity. Glucocorticoids are highly elevated within minutes of the onset of inflammation via activation of the hypothalamus-pituitary-adrenal axis and via release from plasma corticosteroid-binding globulin [7, 8]. It is well known that glucocorticoids elevate blood neutrophil counts [9], expand all developmental stages of neutrophils [10], and inhibit neutrophil apoptosis [11]. These neutrophil-promoting actions of glucocorticoids have been suggested to underlie glucocorticoid resistance of neutrophilic lung inflammation such as that occurs in acute respiratory distress syndrome (ARDS) [12, 13]. In some ARDS patients, glucocorticoid use is even associated with increased mortality [13].

Granulocyte-colony stimulating factor (G-CSF) is one of the most important cytokines that promote neutrophil differentiation, trafficking into circulation, recruitment to tissues, and survival [14]. G-CSF has been recognized as one of the main culprits in neutrophil-driven glucocorticoid-resistant diseases such as ARDS [15–20]. Although it has been known for some time that glucocorticoids can increase G-CSF [21–25], the impact of glucocorticoid-induced G-CSF on neutrophilic lung inflammation has not been examined. We found that blocking G-CSF signaling abolished the protection of neutrophils by glucocorticoids *in vivo*. Glucocorticoids and proinflammatory stimuli synergistically induced G-CSF in BEAS 2B bronchial epithelial cells, A549 lung epithelial cells, human monocyte-derived macrophages, and human neutrophils *in vitro*. Furthermore, the ability of BEAS 2B cells to enhance neutrophil survival was lost when G-CSF shRNAs were expressed in BEAS 2B cells. Overcoming glucocorticoid protection of neutrophils may provide a novel approach to enhance the antiinflammatory actions of glucocorticoids in neutrophil-driven inflammatory conditions.

## Materials and methods

### Animal study approval

This study was carried out in strict accordance with the recommendations in the Guide for the Care and Use of Laboratory Animals of the National Institutes of Health. The protocol was approved by the Institutional Animal Care and Use Committee of Northwestern University (Protocol Approval Number: IS00001650). Husbandry and Veterinary Care of Animals: The Northwestern University animal facility is accredited by the American Association for the Accreditation of Laboratory Animal Care (AAALAC). This facility was responsible for veterinary care programs, operation of the animal facility and assuring that all animal-related procedures meet government standards. Veterinary care programs and operation of the animal facility are directed by veterinarians qualified in Laboratory Animal Medicine. The facility also regularly performs microbiological, parasitological, serological and histological examinations of sentinel mice housed in each animal housing room. Anesthetics: isoflurane was used before intratracheal challenges according to approved protocol. Any unnecessary pain, discomfort, or injury to animals was avoided. Euthanasia: For experiments examining BAL and lungs, the animals were weighed on a scale and euthanized by sodium pentobarbital (100 mg/kg BW) via i.p. injection. Bilateral thoracotomy and removal of vital organs were performed to ensure death.

### Reagents

Recombinant cytokines were from R&D Systems (Minneapolis, MN). Lipopolysaccharide (LPS) was from InVivogen (San Diego, CA). Dexamethasone (DEX) and all other reagents were obtained from Sigma (St. Louis, MO), unless otherwise specified.

### LPS-induced lung injury model

**Fig 1:**
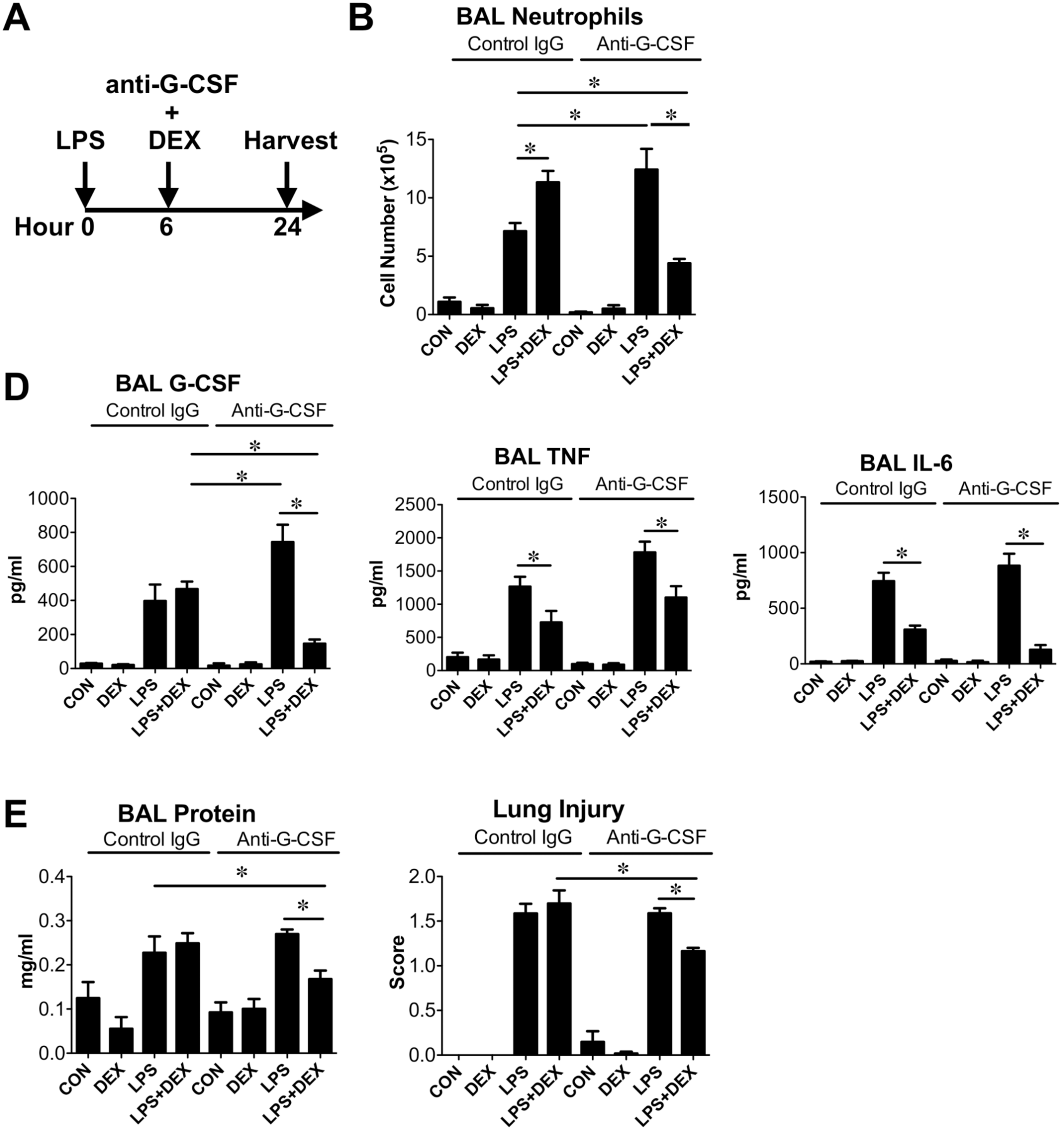
DEX reduced LPS-induced lung inflammation in the presence of G-CSF blockade. **(A)** A diagram depicting the experimental regimen. DEX (2.5 mg/kg, i.p.) and control antibodies or anti-G-CSF (250 μg/kg, i.p.) were given 6 h post LPS (1 mg/kg, i.t., 24 h) challenge. **(B)** BAL neutrophil numbers. ✽, significantly different, two-way ANOVA followed by Bonferroni post-hoc tests. *P*<0.05 (*N* = 4–8). Comparisons indicated are among LPS and LPS+DEX groups in the presence of control or anti-G-CSF antibodies. **(D)** BAL G-CSF, TNF, and IL-6 protein levels. ✽, significantly different, two-way ANOVA followed by Bonferroni post-hoc tests. *P*<0.05 (*N* = 4–8). **(E)** BAL total protein levels and lung injury scores. ✽, significantly different, two-way ANOVA followed by Bonferroni post-hoc tests. *P*<0.05 (*N = 4–*6).

Balb/c mice were obtained from Jackson Lab (Bar Arbor, ME) and bred at Northwestern University. In a previous report, we found that neutrophils are recruited to the lungs of Balb/c mice in a chronic lung inflammation model [26]. Six to eight weeks old male mice were challenged with LPS (1 mg/kg in 100 μl PBS, Invivogen, San Diego, CA) or vehicle intratracheally (Fig 1A). DEX (2.5 mg/kg, i.p., in 100 μl PBS) or vehicle was given 6 h post LPS challenge, to simulate scenarios where lung injuries occur prior to medical intervention. Anti-G-CSF antibodies (Monoclonal Rat IgG1 Clone # 67604, 250 μg/kg in 100 μl PBS, R&D Research) or control antibodies (Rat IgG1, Clone #43414, R&D Research) were given 6 h post LPS challenge. Bronchoalveolar lavages (BALs) and lungs were obtained 24 h after the LPS challenge. In additional experiments, DEX was given 1 h prior to LPS challenge and analyses were performed 3, 6, 12, 24, and 48 h after LPS challenge. BAL total and differential cell counts were determined by flow cytometry and SPHERO AccuCount particles (Spherotech, Inc., Lake Forest, IL). BAL supernatant was frozen at -80°C for ELISA. BAL protein levels were assayed using Bradford method. Before harvesting the lungs, pulmonary vasculature was flushed clear of blood with PBS through right ventricle. The left lung lobe was fixed for histological staining. Hematoxylin and eosin (H&E)-stained lung sections were imaged on an Olympus IX71 microscope using a 10× objective at room temperature. The top three lobes were frozen in liquid N2 and stored at -80°C for homogenate cytokine assays.

### Lung injury scoring

H&E-stained lung sections were scored for presence of cellular inflammation and perivascular infiltration as follows: Grade 0: normal appearance, negligible damage; Grade 1: mild moderate interstitial congestion and neutrophil leukocyte infiltrations; Grade 2: perivascular edema formation, partial destruction of pulmonary architecture, and moderate cell infiltration; Grade 3: moderate lung alveolar damage and intensive cell infiltration; Grade 4: severe cell infiltration and severe destruction of the pulmonary architecture. The score for each lung was calculated from the average score of 10 areas chosen at random.

### Flow cytometry analysis

Rat and hamster normal sera and anti-mouse CD16/CD32 (clone 93) were used to block background signals. Surface staining of single cell suspensions was performed at 4°C for 30 min using the following antibodies, armenian hamster anti-mouse CD11c FITC (N418) from eBiosciences (San Diego, CA), rat anti-mouse CD11b PE (M1/70, eBiosciences), CD45 PE-Cy7 (30-F11A, eBiosciences), Siglec-F (9c7) Alexa Fluor647, ly6G Alex Fluor700 (1A8) from Biolegend (San Diego, CA). Neutrophils were identified as DAPI-CD45+CD11b+siglec-F-ly6G+ and analyzed using a BD LSRII flow cytometer (Franklin Lakes, NJ). Annexin-V and DAPI labeling was processed using the annexin apoptosis detection kit (eBiosciences) according to the manufacturer's instructions. A total of 1.5 x 10^5^ control or treated cells were processed and 1 X 10^4^ cells were analyzed using a BD LSRII flow cytometer (Franklin Lakes, NJ).

### *In situ* hybridization

The bottom right lung lobe was fixed in 4% paraformaldehyde for 12 h, dehydrated using 20% sucrose in 0.12 M KPBS overnight, and store at -80°C. Cryosections of 15 um thick were obtained and vacuum dried overnight before storing in -80°C. For i*n situ* hybridization, sections were postfixed in 4% paraformaldehyde for 15 min at room temperature, incubated in 100 mM Tris and 50 mM EDTA (pH 8.0), 1.32% triethanolamine (pH 8.0), and 0.25% acetic anhydride in 1.32% triethanolamine (pH 8.0) for 10 min at room temperature each. After washing with 2X SSC buffer (300 mM NaCl, 30 mM sodium citrate, pH 7.0) and equilibrating in hybridization buffer buffer (200 mM NaCl, 5 mM EDTA, 10 mM Tris, 5 mM NaH_2_PO_4_, 5 mM Na_2_HPO_4_, 50% deionized formamide, 0.1 mg/ml RNase free tRNA, 10% dextran sulfate (average molecular weight 500, 000), 1X Denhardt’s solution, 50 mM DTT), sections was incubated with the anti-sense mouse G-CSF RNA probe (1:1000) at 58°C for 12 h in a humidified chamber. The DNA sequence for the probe was cloned from balb/c spleen cDNA using primers 5’ GTTGACCAGAGCAAGGGATATAA and 5’ CTGGAGCAAGTGAGGAAGAT and the pcDNA vector (ThermoFischer). RNA probe synthesis and labeling were performed using T7 polymerase and Dig-11-UTP. Probe size (886 bp) and integrity were verified using electrophoresis. After hybridization, slides were washed with 2X SSC at 58°C for 1 h and at room temperature for an additional hour. After blocking with 2% blocking reagent and 10% heat inactivated normal sheep serum (Jackson Immuno, West Grove, PA) in 100 mM malic acid, 150 mM NaCl, and 0.1% Tween 20 (pH 7.5), slides were incubated with anti-Dig-AP Fab (1:1000) over night at 4°C. After extensive washing, AP signals were developed using 1 mg/ml NBT and 0.05 mg/ml BCIP in 100 mM Tris, 100 mM NaCl, and 10% polyvinyl alcohol (average molecular weight 100, 000, pH 9.8) for 24 h at 37°C. After washing with water and dehydration, sections were coverslipped and imaged as described above. Additional slides were processed as above except using the sense strand of the probe.

### ELISA

Cytokine levels in BAL and lung homogenate supernatants were measured using ELISA kits and Super AquaBlue ELISA substrate (eBiosciences). ELISA kits were from R&D Systems (Minneapolis, MN) or BD Pharmingen (San Diego, CA).

### Cell culture

BEAS 2B cells (ATCC, Manassas, VA) were cultured in DMEM-F12 medium (ThermoFisher Scientific, Waltham, MA) containing 5% charcoal-dextran stripped US defined fetal bovine serum (FBS, ThermoFisher), 2 mM glutamine, 50 U/ml penicillin, and 50 μg/ml streptomycin in a 5% CO2 atmosphere at 37°C. Cells were treated as indicated.

### Neutrophil isolation and culture

For human neutrophil preparation, written informed consent for blood donation using a Northwestern University IRB-approved protocol was obtained before enrollment. Neutrophils were purified from EDTA-anticoagulated peripheral blood after density-gradient centrifugation using Percoll (Pharmacia, Uppsala, Sweden) for separation of mononuclear cells from granulocytes, followed by erythrocyte hypotonic lysis and immunomagnetic positive selection with CD16 microbeads (Miltenyi Biotec, San Diego, CA). CD16+ neutrophils cell purity and viability were consistently higher than 98%. Cells were cultured at 1.5 million cells/ml of complete RPMI medium (ThermoFisher Scientific, Waltham, MA) containing 5% charcoal-dextran stripped US defined fetal bovine serum (FBS, ThermoFisher), 2 mM glutamine, 50 U/ml penicillin, and 50 μg/ml streptomycin in a 5% CO2 atmosphere at 37°C. Cells were treated with vehicle, DEX (100 nM, 24 h), and inflammatory stimuli as indicated. For co-culture experiments, cells were incubated together with 2 X 10^5^ BEAS 2B cells on 24-well plates.

### Transduction of BEAS 2B cells

Retrovirus expressing human G-CSF short hairpin (sh) RNA (DNA/RNA delivery core at Northwestern University) and control virus using the MSCV-LTRmiR30-PIG vector (GE Dharmacon, Lafayette, CO) were generated and used to transduce BEAS 2B cell. Positive cells were selected using 1 μg/ml puromycin and verified using realtime RT-PCR.

### Real-time RT-PCR

RNA samples were extracted using the Quick-RNA Miniprep Kit (Zymo Research, Irvine, CA) and treated with DNase according to the manufacturer’s protocol. The level of specific mRNA in each sample was measured using the one-step RT-PCR procedure on a Prism 7500HT thermocycler (Applied Biosystems, Foster City, CA). Primers and probes are 5’ TTCCTGCTCAAGTGCTTAGAG, 5’ AGCTTGTAGGTGGCACAC, and 5’ CATCGCCCTGGATCTTCCTCACT for G-CSF and 5’ TGCAGCTGATCAAGACTGGAGACA, 5’ TCCAGGAAGCGAGAATGCAGAGTT, and 5’ AAGCCACGCTGCTGAACATGCTCAACAT for RPLP0. RPLP0 mRNA was used as the housekeeping gene to normalize the values of G-CSF. Each experiment was performed with at least three biological replicates. Quantification was achieved using the Sequence Detection Software 2.0 Absolute Level subroutine (Applied Biosystems).

### Statistical analysis

Prism software (GraphPad, San Diego, CA) was used. For comparisons of three or more treatment groups, one-way ANOVA was performed followed by Newman-Keuls post-hoc tests. For comparisons with two or more variables, two-way ANOVA was performed followed by Bonferroni post-hoc tests. A *P* value < 0.05 was considered significant. Sample means and SEM are presented.

## Results

### DEX increased LPS-induced airway neutrophil numbers

In a murine acute lung injury model (Fig 1A), neutrophils in the BAL was increased after LPS challenge (Fig 1B). This LPS-induced airway neutrophils were enhanced by the addition of DEX (Fig 1B). LPS also elevated BAL G-CSF, TNF, and IL-6 while addition of DEX decreased TNF and IL-6 (Fig 1C). In striking contrast, LPS-induced BAL G-CSF was not decreased by the addition of DEX (Fig 1C). Reflecting the BAL neutrophil numbers, LPS-induced BAL total protein content and lung injury were not suppressed by DEX (Fig 1D). Because we aimed to determine the role of G-CSF in this model using G-CSF neutralizing antibodies, the experiments described above were performed in the presence of control antibodies.

In additional time course experiments (S1 Fig), we found that DEX enhanced LPS-induced BAL neutrophils also at 14 h, but not at 3, 6, or 48 h after LPS challenge. At all time points examined, DEX inhibited LPS-induced BAL TNF and IL-6 but not G-CSF.

**Fig 2:**
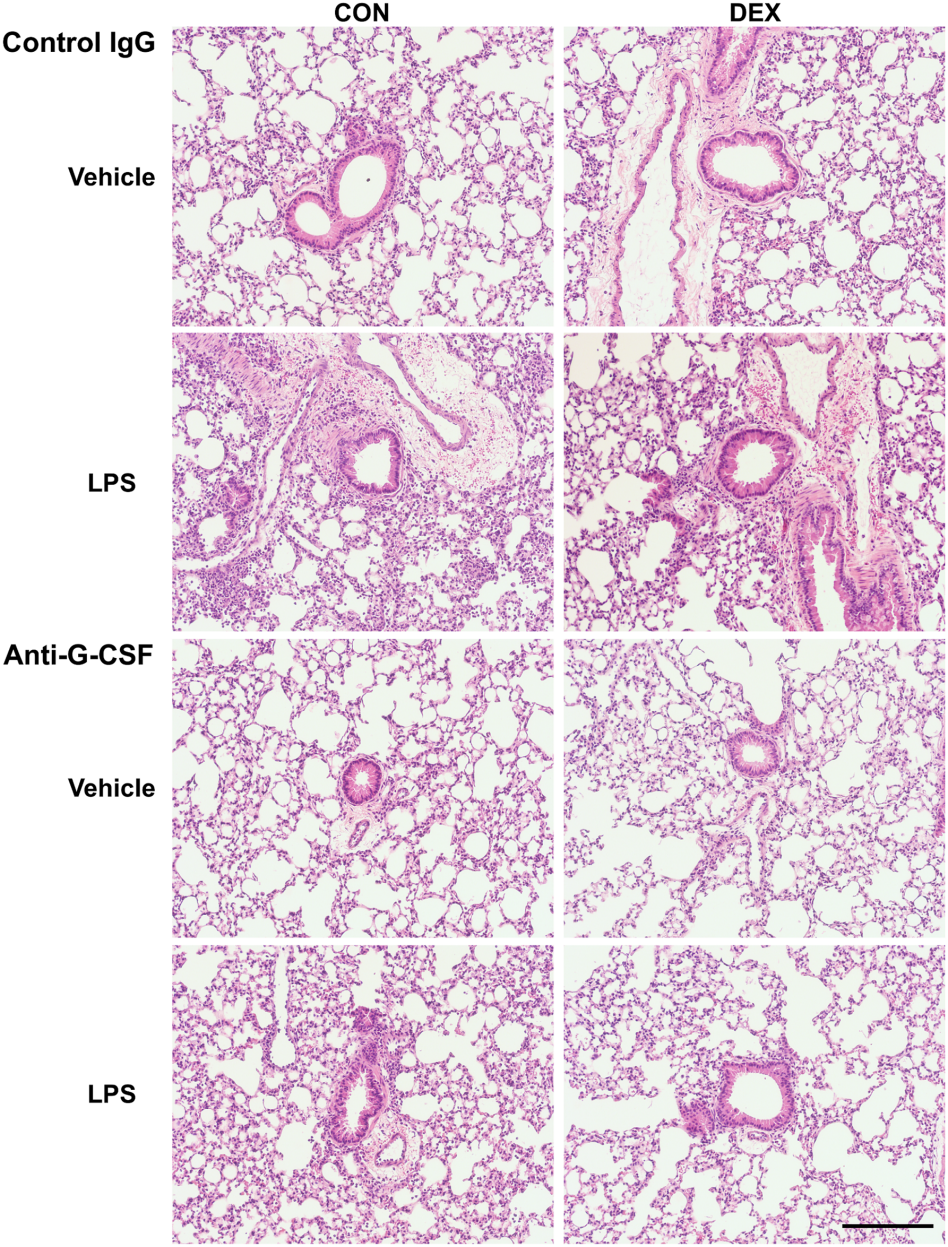
Representative histology of lung sections from different treatment groups. Scale bar, 2 mm.

Since LPS-induced G-CSF was not decreased by DEX, we tested whether the addition of G-CSF neutralizing antibodies to DEX treatment can inhibit BAL neutrophil numbers in LPS-challenged mice (Fig 1). Anti-G-CSF antibodies alone increased LPS-induced BAL neutrophil numbers and G-CSF levels, which has been reported to be due to the ability of anti-G-CSF antibodies to disinhibit CXCR2 signaling [27]. In addition, anti-G-CSF antibodies alone did not inhibit TNF or IL-6, which both contribute to the rise of BAL neutrophils [28, 29]. However, compared to the effects in animals receiving control antibodies, DEX together with anti-G-CSF neutralizing antibodies reduced BAL neutrophil numbers and G-CSF levels in LPS-challenged mice. The ability of DEX to decrease LPS-induced BAL TNF and IL-6 was also maintained in the presence of anti-G-CSF antibodies. Importantly, DEX plus anti-G-CSF antibodies decreased the lung injury score in LPS challenged animals. Representative lung sections from different treatment groups indicating the extent of lung injury are shown in Fig 2.

Furthermore, we measured the G-CSF level in lung homogenates, which reflected the observations in BAL (S1E Fig). In contrast, plasma G-CSF level were marginally elevated in LPS-challenged mice, which was not changed by the addition of DEX (S1 Fig).

### G-CSF was expressed in LPS-challenged murine lungs

**Fig 3:**
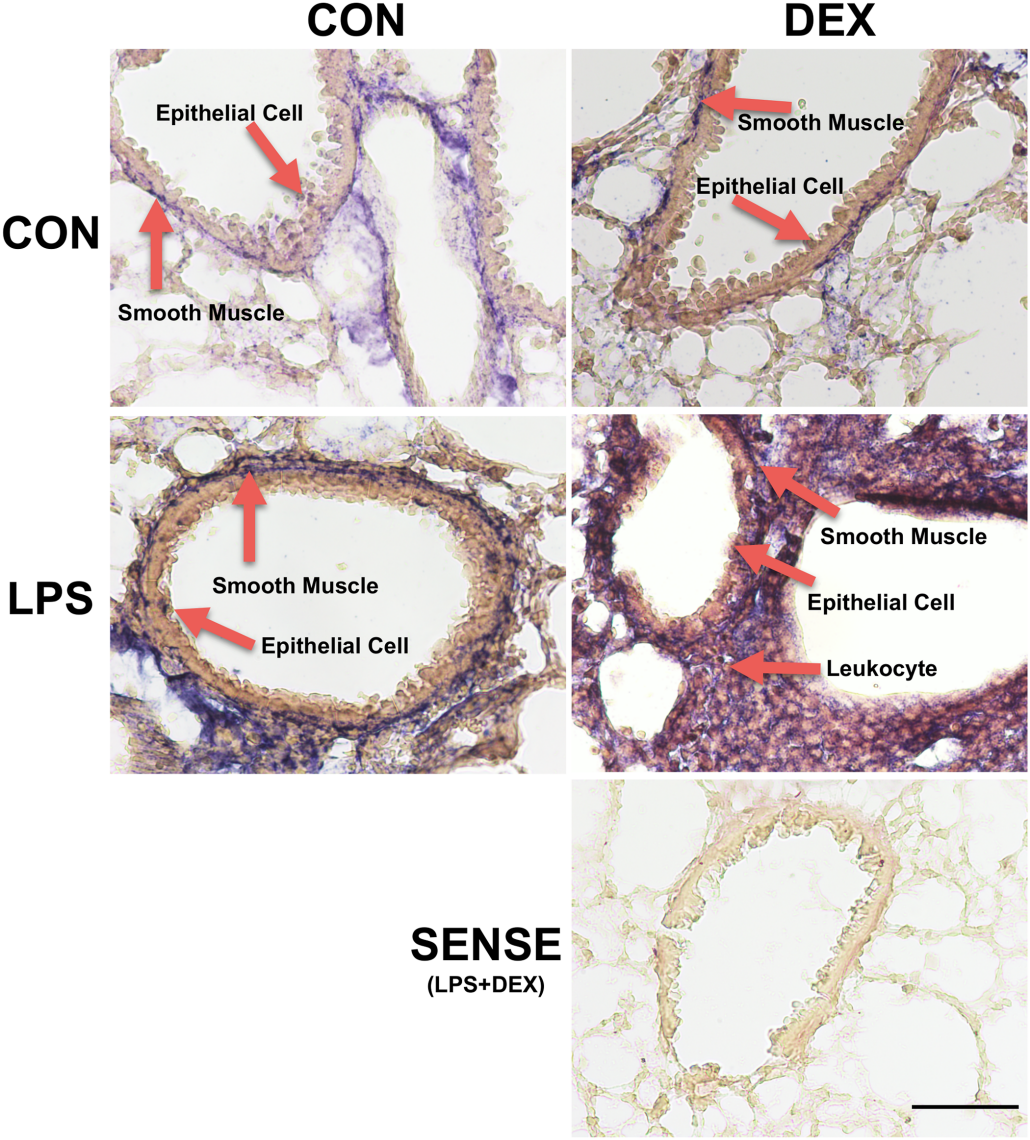
Representative *in situ* hybridization results on lung sections. *In situ* hybridization using a mouse G-CSF anti-sense RNA probe detected G-CSF production locally in lungs of animals treated with vehicle (CON), dexamethasone (DEX, 2.5 mg/kg, i.p., 18 h), LPS (1 mg/kg, i.t., 24 h), and LPS+DEX (6 h post LPS). Cell types with positive signals include smooth muscle cells, epithelial cells, and infiltrated leukocytes (arrows). Control hybridization slides were processed using the sense strand of the RNA probe. Scale bar, 1 mm.

In the LPS-induced acute lung injury model, we found that epithelial cells, smooth muscle cells, and infiltrating leukocytes were producers of G-CSF in lungs using *in situ* hybridization (Fig 3). This observation corroborates previous reports demonstrating that multiple cell types including osteoblasts [30], fibroblasts [31], epithelial cells [32], and endothelial cells [33] all secrete G-CSF.

### DEX and proinflammatory stimuli synergistically induced G-CSF in BEAS 2B cells

**Fig 4:**
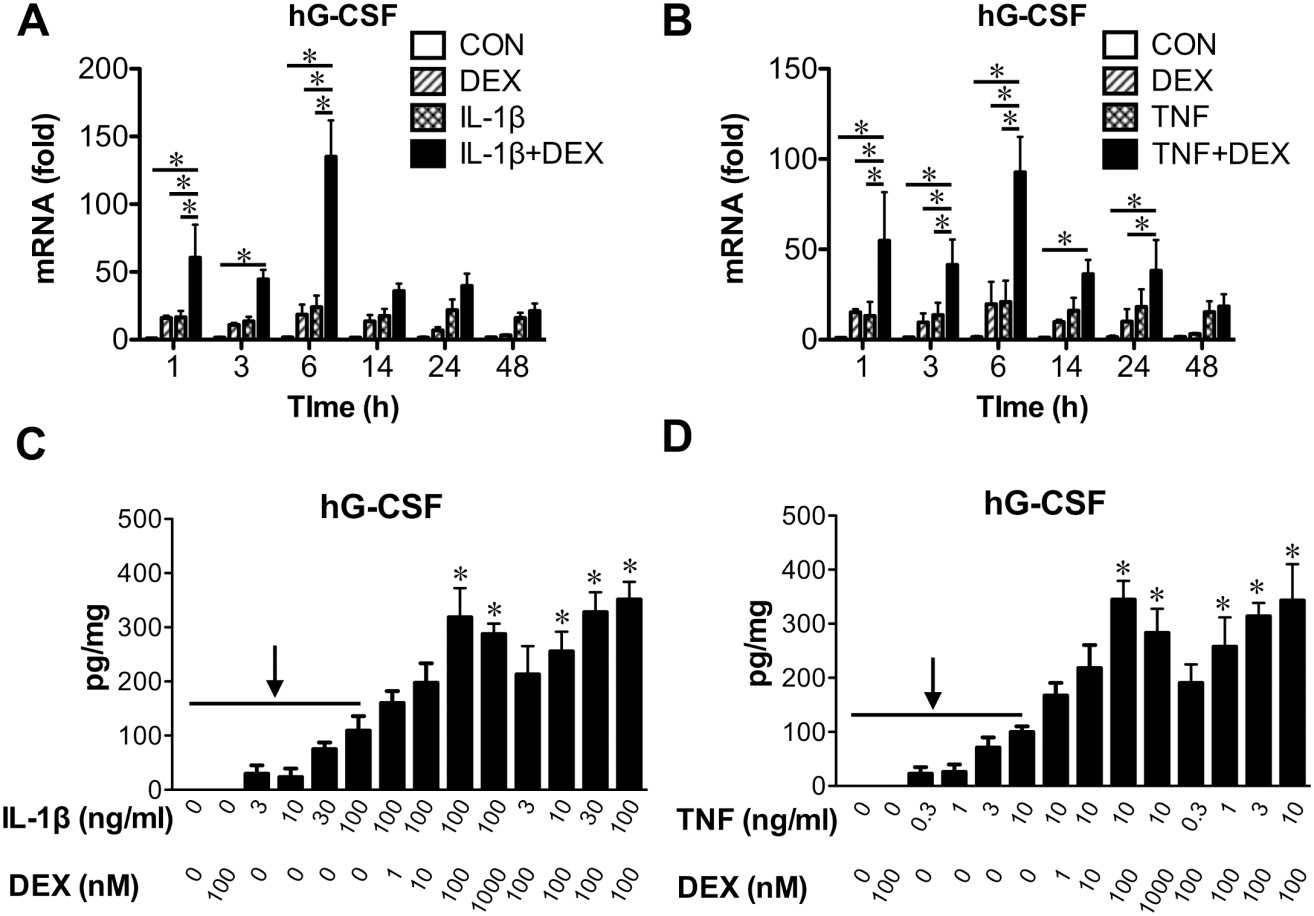
DEX and proinflammatory molecules synergistically induced G-CSF. **(A)** Time course experiment determining IL-1β (10 ng/ml) ± DEX (100 nM) induction of G-CSF mRNA in the immortalized human bronchial epithelial cell line BEAS 2B. Cells were treated in cell culture media supplemented with charcoal-stripped FBS that was devoid of endogenous glucocorticoids. Levels of mRNAs were quantified using realtime RT-PCR and normalized to the level of the housekeeping gene RPLP0. ✽, significantly different, two-way ANOVA followed by Bonferroni post-hoc tests. *P*<0.05 (*N* = 6–10). **(B)** Time course experiment as in (A) using TNF (1 ng/ml) ± DEX (100 nM). ✽, significantly different, two-way ANOVA followed by Bonferroni post-hoc tests. *P*<0.05 (*N* = 6–10). **(C)** DEX (100 nM, 48 h) enhanced IL-1β (3, 10, 30, and 100 ng/ml, 48 h) induction of G-CSF protein in BEAS 2B cell culture supernatant, which was confirmed using various doses of DEX (1, 10, 100, and 1000 nM) with 100 ng/ml IL-1β. ✽, significantly different from the groups indicated by the arrow, one-way ANOVA followed by Newman-Keuls post-hoc tests. *P*<0.05 (*N* = 3). **(D)** Results from dose-response experiments as in (C) using DEX (100 nM) and TNF (0.3, 1, 3, and 10 ng/ml). When using various doses of DEX, TNF was 10 ng/ml. ✽, significantly different from the groups indicated by the arrow, one-way ANOVA followed by Newman-Keuls post-hoc tests. *P*<0.05 (*N* = 3).

Proinflammatory cytokines such as IL-1β and TNF are induced by LPS. DEX and these proinflammatory cytokines (IL-1β and TNF) induced G-CSF mRNA in bronchial epithelial BEAS 2B cells as early as 1 h and the induction persists 6–24 h after treatment (Fig 4A and 4B). A synergy between DEX and IL-1β or TNF in G-CSF mRNA induction was observed at multiple time points. At the protein level, G-CSF was undetectable in the BEAS 2B cell culture supernatant at 24 h post treatment. At 48 h, however, G-CSF was increased by DEX and IL-1β or TNF synergistically (Fig 4C and 4D).

Furthermore, we found TNF and DEX synergistically induced G-CSF expression in human monocyte-derived macrophages, neutrophils, and A549 lung epithelial cells (S2A Fig).

### G-CSF mediates BEAS 2B protection of human neutrophils

**Fig 5:**
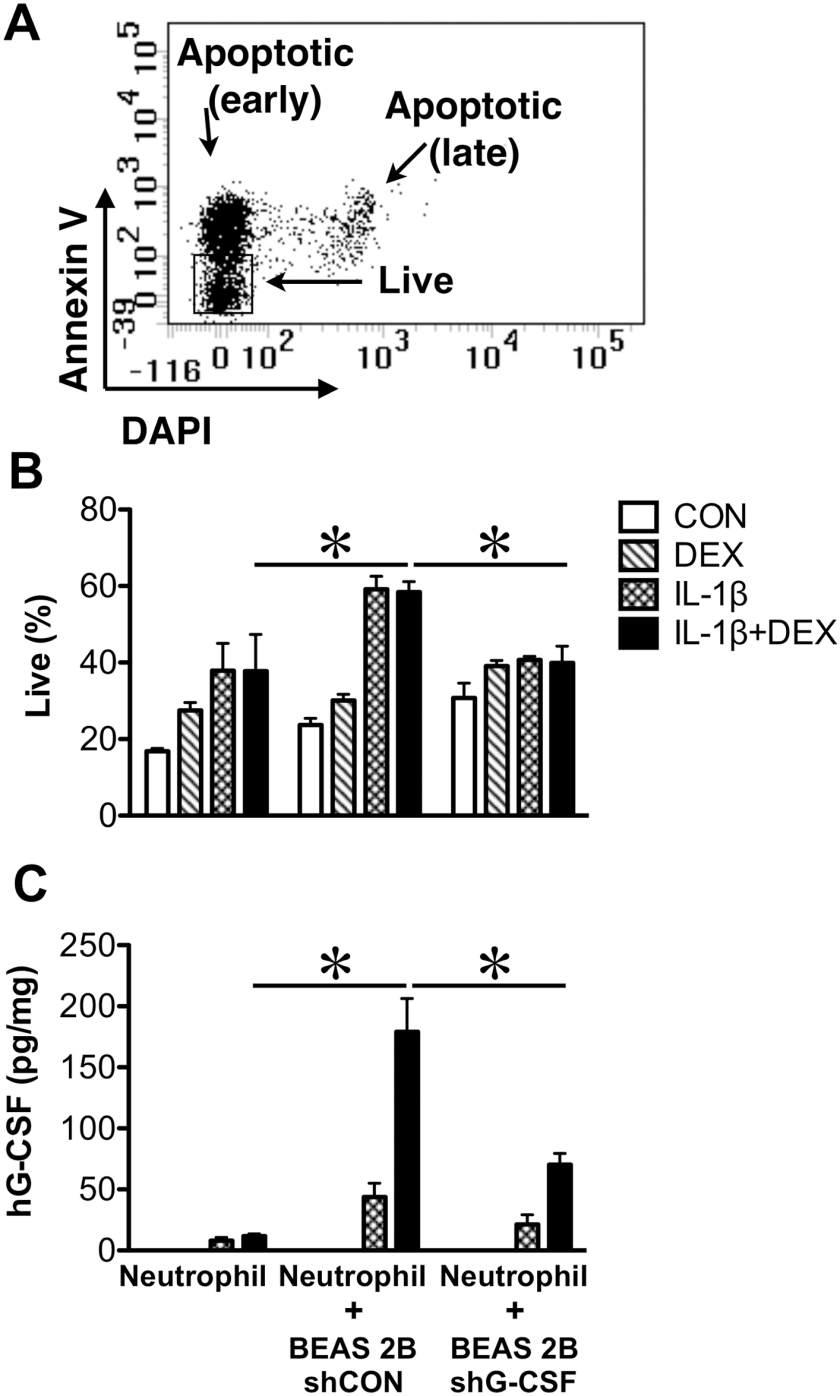
G-CSF induced by glucocorticoid and IL-1β prevents neutrophil apoptosis. **(A)** A representative dot plot of flow cytometric analysis of human neutrophil spontaneous apoptosis 20 h post harvest. Live (annexin V-DAPI-), early apoptotic (annexin-V+DAPI-), and late apoptotic (annexin V+DAPI+) cells were over 97% of the total cells. Necrotic cells (annexin V-DAPI+) were negligible. **(B).** BEAS 2B cells expressing control shRNA (shCON), but not those expressing shRNA for hG-CSF (shG-CSF), protected human neutrophils from spontaneous apoptosis in coculture experiments. BEAS 2B cells or culture media were incubated with vehicle (CON), DEX (10 nM), IL-1β (0.3 ng/ml), or IL-1β+DEX for 24 h before the addition of neutrophils, which were cultured for another 20 h. (C). G-CSF levels in the cell culture media from (B). ✽, significantly different, two-way ANOVA followed by Bonferroni post-hoc tests. *P*<0.05 (*N* = 5). The comparisons shown are between the IL-1β+DEX treated cultures indicated.

To verify the role of G-CSF in neutrophil survival, we examined BEAS 2B and neutrophil cocultures. Human primary neutrophil survival was enhanced by IL-1β and dexamethasone (Fig 5). BEAS 2B cell coculture further protected primary human neutrophils from undergoing apoptosis (Fig 5B). In contrast, this protective effect was lost when G-CSF was knocked down in BEAS 2B cells (Fig 5B and 5C).

In BEAS 2B and neutrophil cocultures, the IL-1β+dexamethasone treatment produced significantly higher amount of G-CSF than IL-1β treatment alone. However, these two treatments protected neutrophils comparably. This observation suggests that BEAS 2B cells, in addition to G-CSF, secrete other neutrophil-protecting molecules, for example CXCL8. We found that IL-1β highly induced CXCL8 expression, which was significantly reduced by dexamethasone (S2B Fig).

## Discussion

Glucocorticoids are beneficial in suppressing GM-CSF, CXCL8, TNF, IL-6, and a wide variety of proinflammatory molecules [34], but paradoxically unable to limit neutrophilic inflammation. Neutrophils, while providing important immunity against pathogens, cause tissue damage via ROS production and protease release [1]. Our findings suggest that blocking G-CSF may provide a potential strategy to increase the anti-inflammatory and anti-neutrophil actions of glucocorticoids (S3 Fig). A major limitation of our studies is the use of *in vitro* cell culture and animal models. Clinical studies are needed to determine the role of G-CSF in ARDS and other neutrophil-dominant diseases. A recent report indicates that therapeutic exercise expedites the recovery of ARDS patients, which has been attributed to a decrease of G-CSF [20].

We observed a discrepancy on DEX regulation of proinflammatory stimulus-induced G-CSF between our *in vivo* and *in vitro* experiments. In BAL, LPS elevated G-CSF levels, which were not changed by the addition of DEX (Fig 1B). In vitro, IL-1β or TNF increased G-CSF production from multiple cell types while addition of DEX further increased G-CSF levels (Fig 4). This discrepancy is potentially due to DEX inhibition of G-CSF-promoting cytokines such as TNF *in vivo*. It has been reported that glucocorticoids increase G-CSF in circulation [21–25], likely via production in mononuclear cells [35]. However, plasma level of G-CSF in LPS-challenged mice were significantly lower than lung G-CSF. In addition, we found multiple cell types in lungs are capable of producing G-CSF. These data support that *in vivo* in our LPS lung injury model, DEX helped to maintain BAL G-CSF while decreasing TNF and IL-6.

Several molecular mechanisms have been suggested to underlie glucocorticoid resistance of neutrophils, including reduced nuclear translocation of glucocorticoid receptor (GR), increased expression of one of the alternatively spliced variants of the GR, the GRβ [36], blunted GR signaling by activation of transcription factors such as NF-κB, and reduced expression of histone deacetylase (HDAC) 2 [37]. However, GR translocation and signaling are intact in neutrophils as demonstrated by the ability of glucocorticoids to elevate the prosurvival MCL-1 and decrease the proapoptotic caspase 8 in neutrophils [38]. Our findings add G-CSF to the repertoire of mediators of glucocorticoid protection of neutrophils. Neutrophils are distributed in homeostasis and inflammation among several locations including bone marrow (> 90% of total neutrophils in homeostasis) [39, 40], blood (1–2% in homeostasis), liver, spleen, and lungs [41] although lungs have been recently called into question as a site for neutrophil margination [42]. Our findings suggest that local production of G-CSF in lungs during inflammation helps to elevate neutrophil numbers in airways, consistent with the published data in bone where local production of G-CSF is necessary for myeloid cell development [30]. IL-17 has been implicated in lung injury [43] and glucocorticoid resistance [44]. Neutrophils help to recruit Th17 cells by secreting CCL20 [45], a chemokine for Th17 cells. CCL-20 is also induced by glucocorticoids [46]. However, BAL IL-17 levels in our LPS model were negligible (data not shown). In a Th17-driven airway inflammation model, we previously reported that glucocorticoids increase lung Th17 cell number and G-CSF [45]. It has been suggested that glucocorticoids strengthen Th17 and neutrophil cross talk during inflammation [47]. Based on these and our research, G-CSF and multiple other factors likely underlie glucocorticoid resistance in neutrophilic inflammation. Early during neutrophil-dominant inflammation, glucocorticoids are beneficial because they suppress a large number of proinflammatory cytokines [47, 48]. Our findings also suggest that long-term glucocorticoid treatment, however, may selectively promote glucocorticoid-resistant cytokines and glucocorticoid-resistant diseases.

## Supporting information

S1 Fig Time course analyses of LPS-induced lung inflammation and measurement of G-CSF in additional tissues. **(A)** A diagram depicting the experimental regimen. DEX (2.5 mg/kg, i.p.) was given 1 h prior to LPS (1 mg/kg, i.t.) challenge. Analyses were performed at indicated time points after the LPS challenge. **(B)** BAL neutrophil numbers. ✽, significantly different, two-way ANOVA followed by Bonferroni post-hoc tests. *P*<0.05 (*N* = 4). Comparisons indicated are between the LPS and LPS+DEX groups. **(C)** BAL G-CSF, TNF, and IL-6 protein levels. ✽, significantly different, two-way ANOVA followed by Bonferroni post-hoc tests. *P*<0.05 (*N* = 4). **(D)** Plasma G-CSF levels. Two-way ANOVA followed by Bonferroni post-hoc tests. *P>*0.05 (*N* = 4). **(E)** Lung homogenate G-CSF levels in animals from Fig 1. ✽, significantly different, two-way ANOVA followed by Bonferroni post-hoc tests. *P*<0.05 (*N* = 4–8).
(TIF)

S2 Fig **(A) DEX and proinflammatory cytokines synergistically induced G-CSF in additional cell types.** Human monocyte-derived macrophages, human neutrophils, and A549 lung epithelial cells were treated with TNF (10 ng/ml) ± DEX (100 nM) for 6 h in cell culture media supplemented with charcoal-stripped FBS that was devoid of endogenous glucocorticoids. Levels of mRNAs were quantified using realtime RT-PCR and normalized to the housekeeping gene RPLP0. **(B) DEX inhibits IL-1β (100 ng/ml) induced CXCL8 in BEAS 2B cells.** ✽, significantly different from other groups, one-way ANOVA followed by Newman-Keuls post-hoc tests. *P*<0.05 (*N* = 3).
(TIF)

S3 Fig A summary of glucocorticoid actions in neutrophilic lung inflammation and the significance of G-CSF. **1)** TNF, IL-1β, and other proinflammatory molecules stimulate the hypothalamus-pituitary-adrenal axis and corticosteroid-binding globulin to release glucocorticoids. **2)** Glucocorticoids exert negative feedback regulation on majority of proinflammatory cytokines. **3)** TNF, IL-1β, and other proinflammatory molecules stimulate G-CSF and lung neutrophilic inflammation. **4)** Proinflammatory molecules and glucocorticoids synergistically stimulate G-CSF and lung neutrophilic inflammation. **5)** Activated lung neutrophils and structural cells produce G-CSF and TNF in a feed-forward loop. **6)** Additional pathways, Th17 for instance, mediate glucocorticoid resistance in neutrophilic inflammation. **7)** Our main finding: Blocking G-CSF, e.g., using anti-G-CSF antibodies, interrupts a major driving force for neutrophilic lung inflammation and may enhance the anti-inflammatory actions of glucocorticoids.
(TIF)

S1 File Supporting methods. (DOCX)
