# Supplementary figures and images for: Granulocyte colony-stimulating factor blockade enables dexamethasone to inhibit lipopolysaccharide-induced murine lung neutrophils
Source: PLoS One. 2017 May 19;12(5):e0177884. doi: 10.1371/journal.pone.0177884 (PMC5438114; doi:10.1371/journal.pone.0177884)

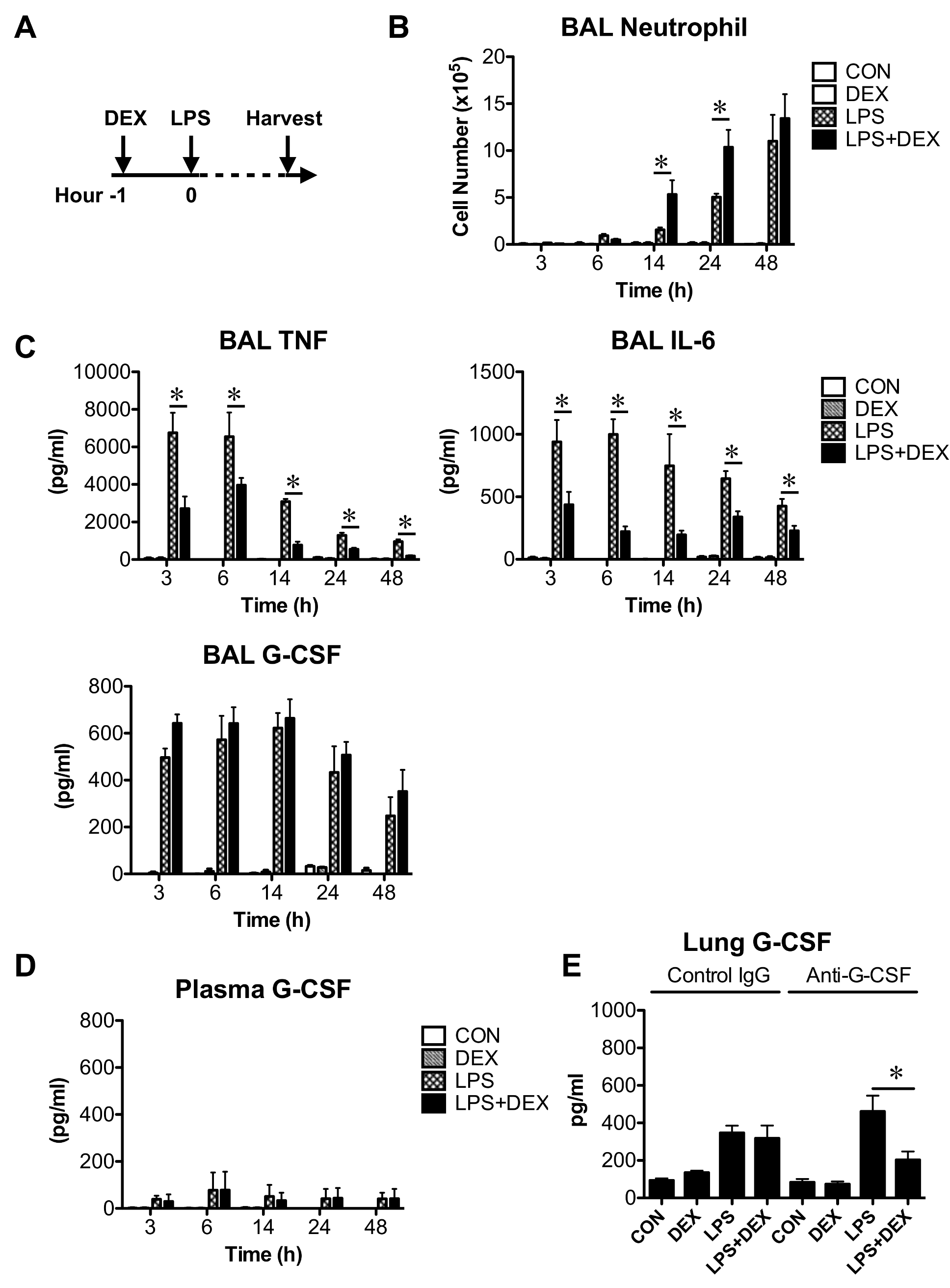

Supplement: S1 Fig — (A) A diagram depicting the experimental regimen. DEX (2.5 mg/kg, i.p.) was given 1 h prior to LPS (1 mg/kg, i.t.) challenge. Analyses were performed at indicated time points after the LPS challenge. (B) BAL neutrophil numbers. ✽, significantly different, two-way ANOVA followed by Bonferroni post-hoc tests. P<0.05 (N = 4). Comparisons indicated are between the LPS and LPS+DEX groups. (C) BAL G-CSF, TNF, and IL-6 protein levels. ✽, significantly different, two-way ANOVA followed by Bonferroni post-hoc tests. P<0.05 (N = 4). (D) Plasma G-CSF levels. Two-way ANOVA followed by Bonferroni post-hoc tests. P>0.05 (N = 4). (E) Lung homogenate G-CSF levels in animals from Fig 1. ✽, significantly different, two-way ANOVA followed by Bonferroni post-hoc tests. P<0.05 (N = 4–8). (TIF) [file pone.0177884.s001.tif]

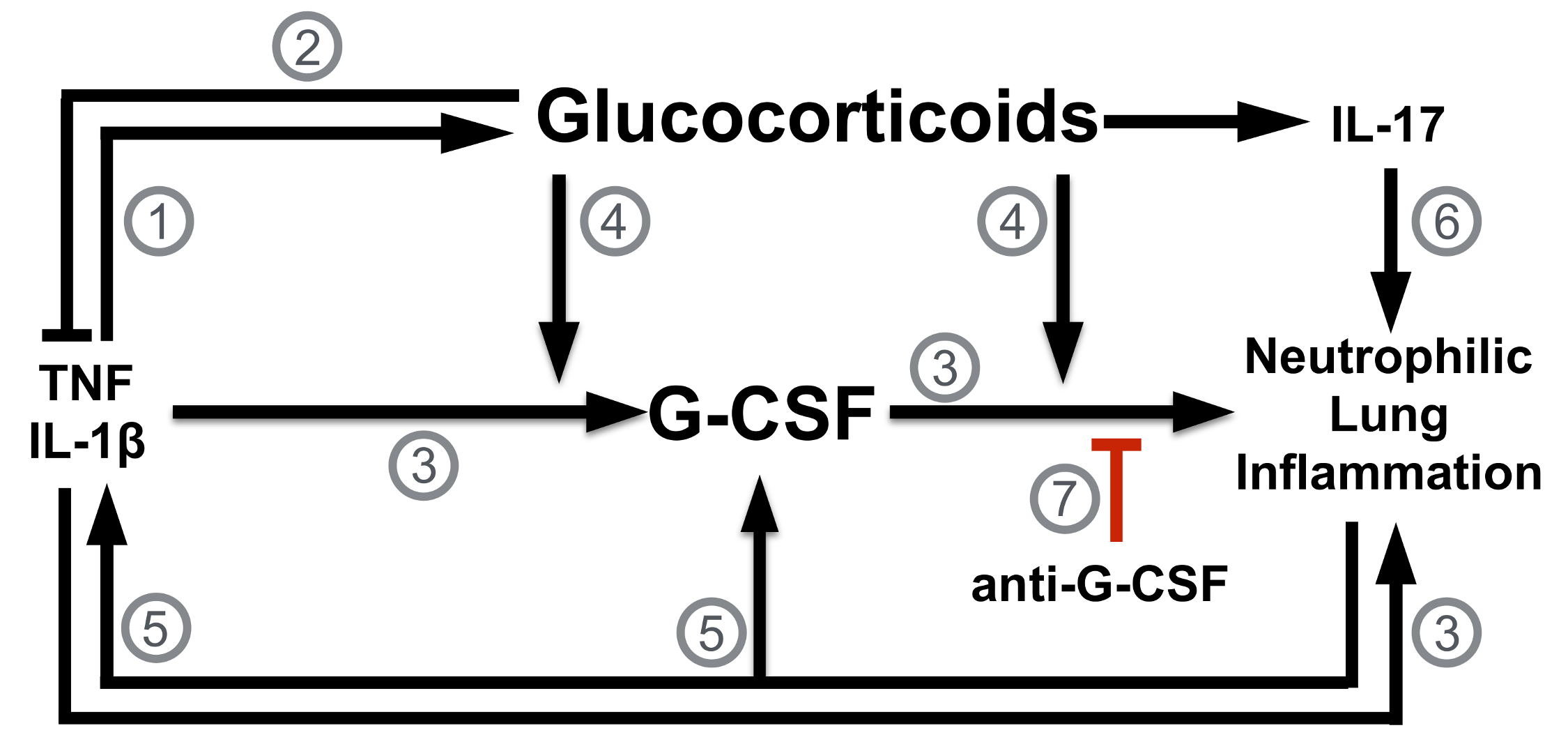

Supplement: S3 Fig — 1) TNF, IL-1β, and other proinflammatory molecules stimulate the hypothalamus-pituitary-adrenal axis and corticosteroid-binding globulin to release glucocorticoids. 2) Glucocorticoids exert negative feedback regulation on majority of proinflammatory cytokines. 3) TNF, IL-1β, and other proinflammatory molecules stimulate G-CSF and lung neutrophilic inflammation. 4) Proinflammatory molecules and glucocorticoids synergistically stimulate G-CSF and lung neutrophilic inflammation. 5) Activated lung neutrophils and structural cells produce G-CSF and TNF in a feed-forward loop. 6) Additional pathways, Th17 for instance, mediate glucocorticoid resistance in neutrophilic inflammation. 7) Our main finding: Blocking G-CSF, e.g., using anti-G-CSF antibodies, interrupts a major driving force for neutrophilic lung inflammation and may enhance the anti-inflammatory actions of glucocorticoids. (TIF) [file pone.0177884.s003.tif]
