# Supplementary material for: Granulocyte colony-stimulating factor blockade enables dexamethasone to inhibit lipopolysaccharide-induced murine lung neutrophils
Source: PLoS One. 2017 May 19;12(5):e0177884. doi: 10.1371/journal.pone.0177884 (PMC5438114; doi:10.1371/journal.pone.0177884)
Supplement: S1 File — (DOCX) [file pone.0177884.s004.docx]

**Supporting Methods**

**Macrophage isolation and culture**

For human macrophage preparation, same donors for neutrophils were recruited. Bone marrow monocytes were adhere to cell culture plates in RPMI medium supplemented with 10% FBS for two hours and differentiated into macrophages using RPMI medium supplemented with 20% L929 conditioned medium for one week. Cells were incubated with RPMI medium supplemented with 5% charcoal-dextran stripped FBS for two days before DEX treatments.

**A549 cell culture**

A549 cells (ATCC, Manassas, VA) were cultured as described for BEAS 2B cells.
